# Compatibility of Fuzi and Ginseng Significantly Increase the Exposure of Aconitines

**DOI:** 10.3389/fphar.2022.883898

**Published:** 2022-04-26

**Authors:** Ze-Yan Chen, Xu-Ya Wei, Zi-Dong Qiu, Yun Huang, Ting Tan, Yu-Lin Feng, Juan Guo, Guang-Hong Cui, Lu-Qi Huang, Chang-Jiang-Sheng Lai

**Affiliations:** ^1^ State Key Laboratory Breeding Base of Dao-di Herbs, National Resource Center for Chinese Materia Medica, China Academy of Chinese Medical Sciences, Beijing, China; ^2^ School of Traditional Chinese Medicine, Guangdong Pharmaceutical University, Guangzhou, China; ^3^ Jiangxi University of Traditional Chinese Medicine, Nanchang, China; ^4^ Pharmaceutical College, Hebei Medical University, Shijiazhuang, China; ^5^ The National Pharmaceutical Engineering Center for Solid Preparation in Chinese Herbal Medicine, Jiangxi University of Traditional Chinese Medicine, Nanchang, China

**Keywords:** Aconitum carmichaelii, ginseng, pharmacokinetics, aconitine, high performance liquid chromatography-mass spectrometry, COVID-19

## Abstract

The herb-pair ginseng-Fuzi (the root of *Aconitum carmichaelii*) is the material basis of Shenfu prescriptions and is popular in traditional Chinese medicine for the treatment of heart failure, and even shock with severe-stage of COVID-19. A narrow therapeutic window of Fuzi may cause significant regional loss of property and life in clinics. Therefore, systemic elucidation of active components is crucial to improve the safety dose window of Shenfu oral prescriptions. A high performance liquid chromatography-mass spectrometry method was developed for quantification of 10 aconitines in SD rat plasma within 9 min. The limit of detection and the limit of quantification were below 0.032 ng/ml and 0.095 ng/ml, respectively. Furthermore, a systemic comparison with their pharmacokinetic characteristics after oral administration of a safe dosage of 2 g/kg of Fuzi and ginseng-Fuzi decoction for 24 h was conducted. Eight representative diester, monoester, and non-ester aconitines and two new active components (i.e., songorine and indaconitine) were all adopted to elucidating the differences of the pharmacokinetic parameters *in vivo*. The compatibility of Fuzi and ginseng could significantly increase the *in vivo* exposure of active components. The terminal elimination half-life and the area under the concentration-time curve of mesaconitine, benzoylaconitine, benzoylmesaconitine, benzoylhypaconitine, and songorine were all increased significantly. The hypaconitine, benzoylmesaconitine, and songorine were regarded as the main active components *in vivo*, which gave an effective clue for the development of new Shenfu oral prescriptions.

## 1 Introduction

Toxic-efficient dual Chinese medicines have remarkable efficacy and certain toxicity or side effects. If used improperly, it will cause unavoidable toxic side effects, and even endanger patients’ lives in serious cases (Wei et al., 2019). Due to the irreplaceability in the potent effects, toxic-efficient dual Chinese medicines are still widely used in clinics. The lateral root of *Aconitum carmichaelii* Debx (named Fuzi) is commonly used for the treatment of rheumatism, heart failure, and renal failure (Wang et al., 2007; Li et al., 2017a; Shuo et al., 2017; Chen et al., 2021). However, it often triggers aconitine poisoning events due to a narrow therapeutic window (Singhuber et al., 2009; Huang et al., 2018; Qiu et al., 2021a). The main active components in Fuzi are aconitines, including diester alkaloids, i.e., aconitine, mesaconitine and hypaconitine, and monoester alkaloids, i.e., benzoylaconitine, benzoylmesaconitine, and benzoylhypaconitine (Qiu et al., 2021a; Qiu et al., 2021b). However, the diester alkaloids are considered to be the main toxic components for the cardiac and central nervous systems. The toxicity of diester alkaloids is 200–500 times and 2000–4,000 times of monoester alkaloids and non-ester alkaloids, i.e., aconine, mesaconine, and hypaconine, respectively (Liu et al., 2017). The cardiotoxicity target of diester alkaloids is the site 2 of sodium channel. Their cardiotoxicity mechanism is a large influx of Na^+^ causes persistent malignant arrhythmias (Chan et al., 1994; Fu et al., 2006; Chen et al., 2013).

Compatibility has been often used to reduce toxicity and increase efficacy (Zhang et al., 2012; Zhang et al., 2013; Liu et al., 2014; Liu et al., 2017; Sun et al., 2018; Qiu et al., 2020a). The pharmacokinetic characterizations of herb-pairs Fuzi-Gancao (Zhang et al., 2013; Zhang H. et al., 2015), Fuzi-ginger (Peng et al., 2013; Zhang W. et al., 2015), Fuzi-Beimu (Yang et al., 2016; Xu et al., 2017), and ginseng-Fuzi (Shenfu) (Li Z. et al., 2015; Yang et al., 2018), and formula [e.g., Wutou Decoction (Dai et al., 2014), Sini Decoction (He et al., 2009; Zhang H. et al., 2015; Zhang W. et al., 2015; Zhou et al., 2019), Dahuang Fuzi Decoction (Liu et al., 2014; Li YX. et al., 2015; Li et al., 2017b), and Shenfu injectable powder (Zhang et al., 2008; Li Z. et al., 2015; Zhang et al., 2016)] have been elucidated (He et al., 2015; Chen et al., 2019; Wei et al., 2019). Currently, the pharmacokinetic studies of aconites were usually reported on Shenfu injection (Zhang et al., 2008; Zhang et al., 2016; Shen et al., 2021). There is no other relevant study with simultaneous quantification of three types of aconitines for oral preparations containing Fuzi-ginseng herb pair (Xu et al., 2020). Shenfu preparations have been commonly used in the treatment of heart failure, and even the shock patient of severe-stage COVID-19. In daily life, the oral drugs are easily accepted by patients and have a promising market prospect in the treatment of chronic heart failure. However, their narrow oral safety dose window makes it difficult to effectively balance cardiac efficacy and cardiotoxicity, resulting in extremely low market share and difficulty in applying new oral drugs. It is urgent to elucidate the *in vivo* active components and compatibility mechanism to develop new oral drugs further.

**FIGURE 1:**
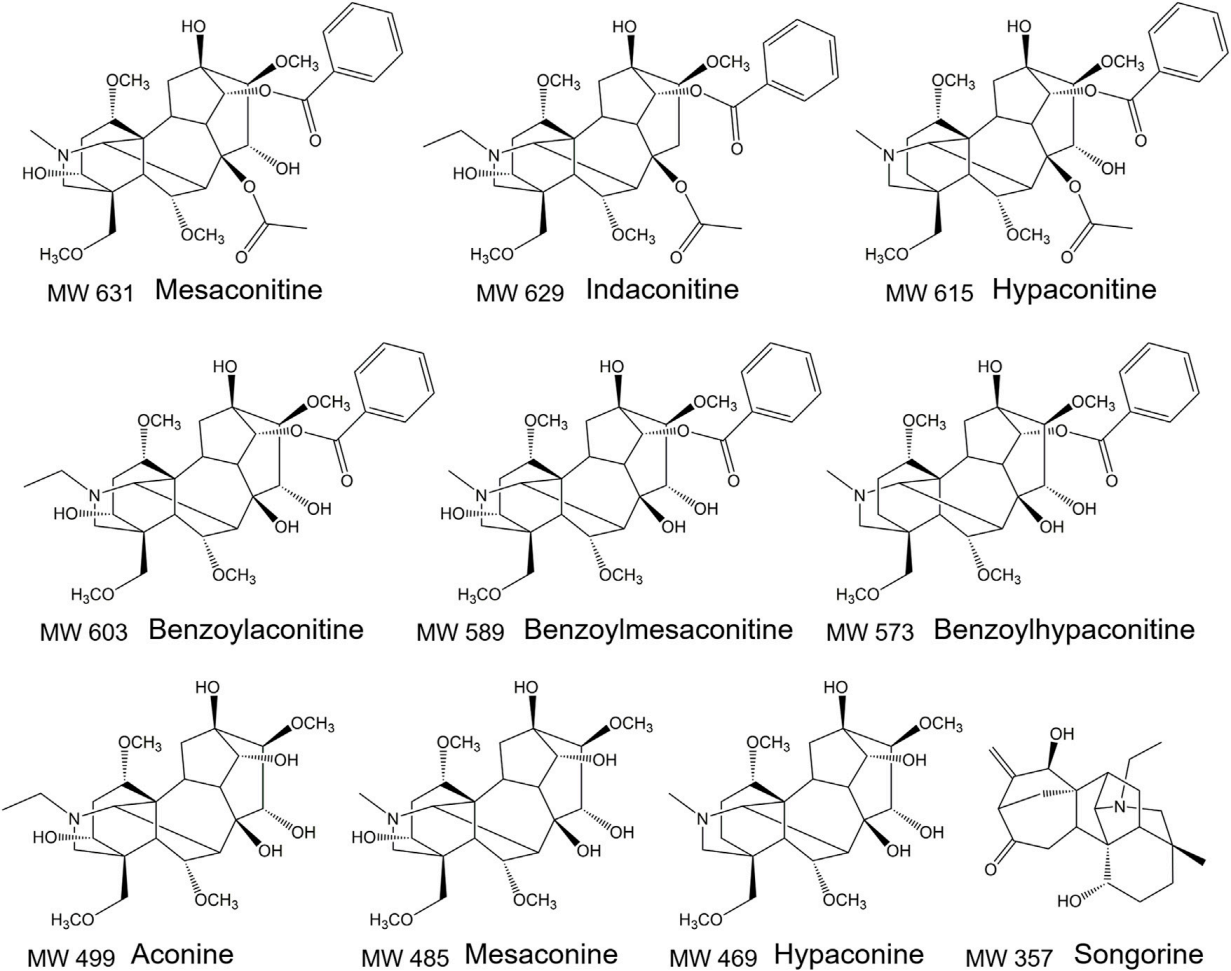
The structure of 10 aconitines.

High performance liquid chromatography coupled with tandem mass spectrometry (HPLC-MS/MS) contains the advantages of high throughput, high sensitivity, and high resolution for analysis of complex matrix samples (Garran et al., 2019). In this study, an HPLC-MS/MS method for quantification of 10 aconitines components with all three types of structure in rat plasma was developed (Figure 1). The method had a lower limit of detection (LOD) and limit of quantification (LOQ) than other methods (Zhang et al., 2014; Xu et al., 2020). The comparative pharmacokinetic study between Fuzi decoction with ginseng-Fuzi decoction was conducted to screen out the *in vivo* active components of Shenfu oral prescriptions. Moreover, the pharmacokinetic parameters of songorine and indaconitine were studied in Shenfu decoction for the first time. The aim is to lay the foundation for the scientific design of the prescription, dosage, and controlled active/toxic combinatorial components of Shenfu oral prescriptions.

## 2 Experimental

### 2.1 Materials and Reagents

Processed Aconitum (Heishunpian) was purchased from Sichuan, China. Ginseng was purchased from Tongrentang Chinese Medicine. The specimens were stored in the National Resource Center for Chinese Materia Medica, Chinese Academy of Chinese Medical Sciences. Berberine (internal standard) was purchased from ANPEL Laboratory Technologies (Shanghai) Inc. (Shanghai, China, purity >98%). Eight authentic components including mesaconitine, indaconitine, hypaconitine, benzoylaconitine, benzoylmesaconitine, benzoylhypaconitine, aconine, and songorine were supplied by Beijing Rongcheng Xinde Technology Development Co., Ltd. (Beijing, China, HPLC purity >98%). Another two authentic components including mesaconine and mesaconine were acquired from Chengdu Must Biotechnology Co., Ltd. (Chengdu, Sichuan, China). The purity of each component was >98%, as determined by HPLC analysis. Pure water was prepared from Mill-Q water purification system (Billerica, MA, United States). Methanol and acetonitrile (HPLC grade) were purchased from ThermoFisher Scientific (San Jose, CA, United States). Ammonium chloride (AR) was purchased from Aladdin Industrial Corporation (Shanghai, China).

### 2.2 Animals

Male Sprague-Dawley (SD) rats (*n* = 12) weighted 180–220 g were supplied by Laboratory Animal Science and Technology Center, Jiangxi University of Traditional Chinese Medicine (Nanchang, Jiangxi, China). Animals were housed under standard conditions for a week of adjustable feeding. All animal experiments were carried out according to the Guidelines for the Care and Use of Laboratory Animals and were approved by the Animal Ethics Committee of Jiangxi University of Traditional Chinese Medicine.

### 2.3 Preparation of Standard Solutions

A series of mixed working solutions at gradient concentrations were prepared by dissolving appropriate amounts of 10 aconitines with methanol and gradient dilution. The frozen plasma samples were thawed naturally at room temperature, 10 μL of mixed working solutions and 90 μL plasma were mixed and vortexed for 1 min with sufficient mixing. The 10 μL of internal standard solution (berberine, 500 ng/ml) and 300 μL of methanol were added. All samples were vortexed at 2,500 rpm for 3 min and centrifuged at 10,000 rpm for 10 min at 4°C. The supernatant was collected and was then dried under nitrogen at 40°C. The 100 μL of methanol was added to redissolve the residue. After vortexing for 1 min, the resolution was centrifuged at 14,000 rpm for 10 min at 4°C, and the supernatant was collected and stored at −20°C until analysis.

### 2.4 Sample Preparation

The 12.5 g of processed Fuzi powder were weighed and soaked for 30 min in water (1:10, *w*/*v*), then was decocted for 30 min. The filtrate through 8 layers of gauze was collected. The residues were re-decocted by 8 times of water for 30 min. The two filtrates were combined and concentrated by rotary evaporator at 40°C to 0.175 g/ml (in terms of Fuzi) of Fuzi extract was prepared, containing mesaconitine 0.03 μg/ml, indaconitine 0.12 μg/ml, hypaconitine 1.04 μg/ml, benzoylaconitine 10.16 μg/ml, benzoylmesaconitine 36.40 μg/ml, benzoylhypaconitine 14.28 μg/ml, aconine 2.04 μg/ml, mesaconine 6.02 μg/ml, hypaconine 2.74 μg/ml, songorine 9.04 μg/mL. As for Shenfu extract, the mass ratio of Fuzi and ginseng was 1:1. The other preparation steps were the same as those of Fuzi extract, containing 0.10 μg/ml of mesaconitine, 4.85 μg/ml of hypaconitine, 11.70 μg/ml of benzoylaconitine, 28.30 μg/ml of benzoylmesaconitine, 12.10 μg/ml of benzoylhypaconitine, 1.71 μg/ml of aconine, 5.31 μg/ml of mesaconine, 1.68 μg/ml of hypaconine, and 6.25 μg/ml of songorine. The quantification of alkaloids was performed according to our validated HPLC-MS/MS method. All extracts were stored at 4°C.

### 2.5 Liquid Chromatography With Tandem Mass Spectrometry Conditions

The Shimadzu LC-30AD (Kyoto, Japan) consisted of a binary pump and a sample manager was applied as the LC system. Gradient elution was performed on a Waters ACQUITY UPLC BEH C18 column (1.7 μm, 2.1 mm × 100 mm) protected by a Van Guard BEH C18 column (1.7 μm, 2.1 mm × 5 mm). The column temperature was maintained at 35°C. The experiment was carried out at a flow rate of 0.4 ml/min. The injection volume was 2 μL. The mobile phase was acetonitrile (solvent B)—water (solvent A) containing 0.5 mM ammonium chloride. Gradient elution was performed as follow: 0–2 min 35% B, 2–4 min 35–85% B, 4–6 min 85–90% B, 6–7 min 90–100% B, 7–9 min 100% B, 9–9.5 min 100–35% B, and 9.5–12.5 min 35% B. QTRAP 4500 mass spectrometer (Applied Bio-systems, AB Sciex, United States) coupled with ESI source was employed in the MS/MS analysis. Mass spectrum parameters were set as follows: Curtain Gas = 35 psi, Collision Gas = Medium, IonSpray Voltage = 4500 V, Temperature = 550°C, and Gas1 = Gas2 = 55 psi. MRM mode was adopted to detect the target components and internal standard (50 ng/ml). The DP and CE were automatically optimized to enhance the intensity of ion pairs of all the target components. All samples were analyzed by LC-MS in positive ion mode.

### 2.6 Method Validation

#### 2.6.1 Specificity

The specificity was investigated by comparing the chromatograms of blank rat plasma, corresponding spiked plasma, and rat plasma sample at 45 min after oral administration of Fuzi, to exclude the interference of endogenous substances and metabolites.

#### 2.6.2 Linearity, Limit of Detection, and Limit of Quantification

For the calibration curve, the gradient dilution was used to obtain a series of solutions with gradient concentrations (0.001–125 ng/ml) for LC-MS analysis. The regression equation and correlation coefficient (*R* ^2^) were calculated using the concentration of the component as the horizontal coordinate (*X*, ng/mL) and the ratio of the integrated peak area of the component to the internal standard as the vertical coordinate (*Y*). The concentration was used as the LOD and the LOQ at the signal-to-noise ratio (S/N) equal to 3 and 10, respectively.

#### 2.6.3 Precision and Stability

The QC samples of high, medium, and low concentrations were injected six times consecutively and replicated for three consecutive days, and the intra-day precision and precision were calculated and expressed as relative standard deviation (RSD). The stability assay of the high, medium, and low concentrations of mixed standards in plasma samples was conducted. All prepared samples were stored for 12 h at room temperature to evaluate their room temperature stability. As for freeze-thaw stability, the plasma samples were stored for 12 h at room temperature and then 12 h at −20°C, and repeated three times. The plasma samples of the long-term stability analysis should be stored for 15 days at −20°C. All samples were injected under the same conditions for LC-MS analysis and their mean concentration, standard deviation (SD), and RSD were calculated.

#### 2.6.4 Recovery and Matrix Effect

The pre-extraction samples were prepared according to the preparation of standard solutions. The blank plasma was prepared with the same method, and then 10 μL of 500 ng/ml internal standard solution and 90 μL of mixed standard solution were added to redissolve the residue. These samples were recorded as post-extraction samples. All samples were analyzed by the same LC-MS conditions and the extraction recoveries were calculated according to formula 1.

$$Extraction\;re\mathrm{cov}ery\;\%=\frac{A_{pre-extraction\;sample}/A_{internal\;standard}}{A_{post-extraction\;sample}/A_{internal\;standard}}$$ (1)

The mixed working solutions were prepared in methanol with high, medium, and low concentrations respectively and analyzed by the same LC-MS conditions. The matrix effect was calculated according to formula 2.

$$Matrix\;effect\;\%=\frac{A_{post-extraction\;sample}/A_{internal\;standard}}{\;A_{mixed\;solution}/A_{internal\;standard}}$$ (2)

### 2.7 Pharmacokinetics

Male SD rats were randomly divided into two groups of six rats each for Fuzi and Shenfu groups. The animals were acclimatized and fed for 7 days. Before the experiment, the animals fasted for 12 h without water. The animals were administered by the same dosage (equal to 2 g/kg of Fuzi). The blood was collected into heparinized tubes before (0 h), 0.25, 0.5, 0.75, 1, 1.5, 2.5, 4, 6, 8, 12, and 24 h after administration, and centrifuged at 4,000 rpm for 10 min at 4°C. 100 μL of supernatant was obtained and stored at −80°C before analysis. The corresponding peak area integration values were recorded. The concentration was calculated using the corresponding calibration equation.

### 2.8 Statistical Analysis

The drug concentration-time curve was plotted using time as the horizontal coordinate and the mean value of the blood concentration corresponding to each time point as the vertical coordinate. The relevant pharmacokinetic parameters, including terminal elimination half-life (*T*_1/2_), area under the concentration-time curve (*AUC*_0-t_), mean residence time (*MRT*_0-t_), time to achieve maximum concentration (*T*_max_), and maximum plasma concentration (*C*_max_), were calculated using non-compartment analysis with DAS software and expressed as mean ± standard deviation. The relative bioavailability was calculated by formula 3. The comparison of the main pharmacokinetic parameters between the Fuzi group and the Shenfu group was performed by independent samples t-test with SPSS software.

$$Relative\;bioavailability\left(\;F_{rel},\;\%\right)=\frac{AUC_{0-t,\;Shenfu}}{AUC_{0-t,\;Fuzi}}$$ (3)

## 3 Result and Discussion

### 3.1 Optimization of High Performance Liquid Chromatography Coupled With Tandem Mass Spectrometry Conditions

**FIGURE 2:**
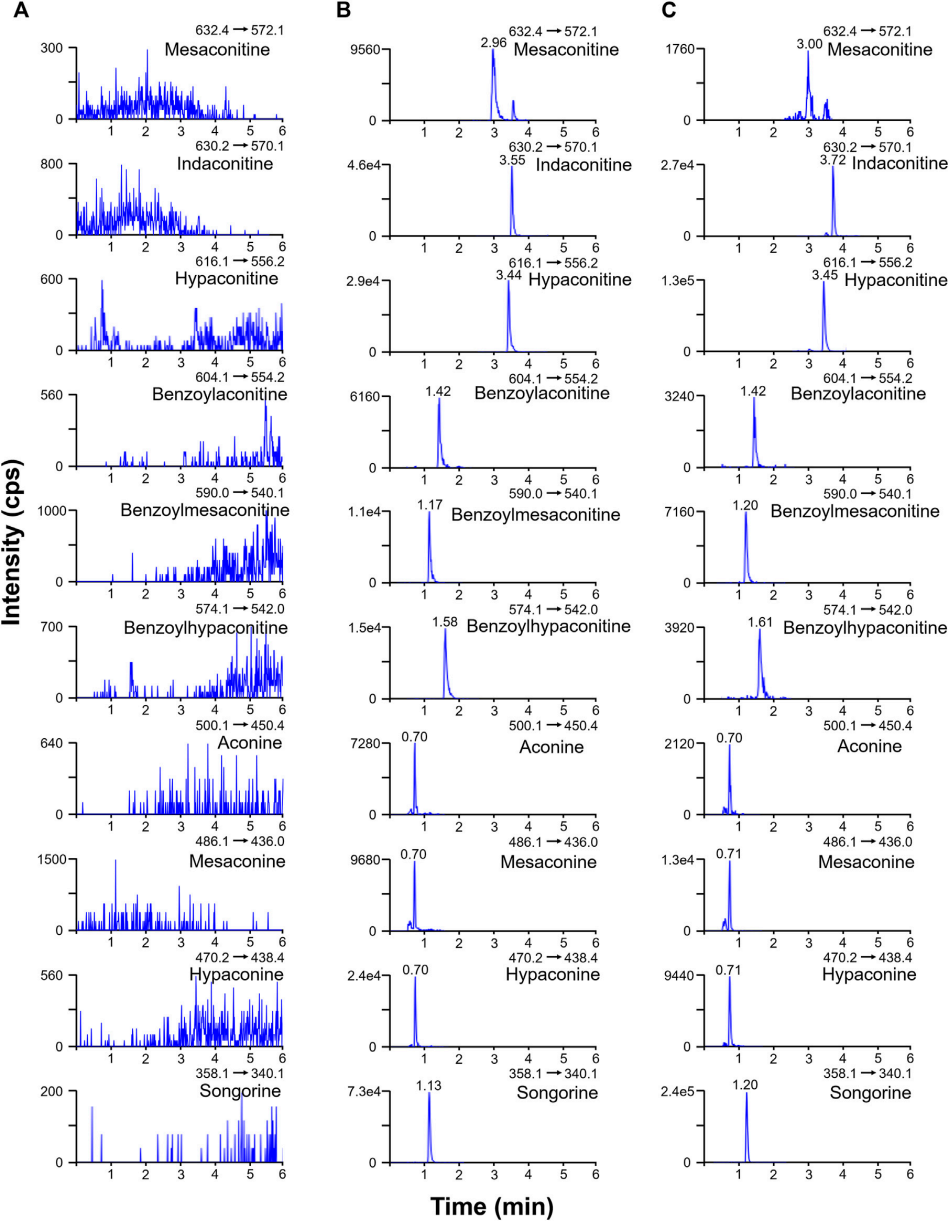
Multiple reaction monitoring chromatograms of blank plasma **(A)**, spiked standard solution in blank plasma **(B)**, and the rat plasma sample at 45 min after oral administration of Fuzi **(C)**.

The ion spray voltage (3.5–5.0 kV) and source temperature (350–550°C) were optimized in the positive ion mode to determine the mass spectrometry conditions in terms of the response intensity and noise intensity of the components. The final mass spectrometry conditions were determined as follows: positive ion mode Curtain Gas = 35 psi, Collision Gas = Medium, Ion Spray Voltage = 4500 V, Temperature = 550°C, Gas1 = Gas2 = 55 psi. The ESI-MS was injected at a flow rate of 7 μL/min and the optimized mass spectrometry conditions were used for the analysis. The collision energy (CE) and declustering potential (DP) were automatically optimized by the instrument. The final ion pairs and related parameters were determined as Table 1. The 0.5 mM ammonium chloride in the study had no significant inhibitory effect on aconitines with LOD below 0.03 ng/ml. Column temperatures were optimized at 30, 35, 40, and 45°C, and small differences were found. To avoid degradation of aconitines, which are easily decomposed by heat, during the analysis and resulting in reduced accuracy, 35°C was chosen as the analytical column temperature in this experiment and the sample tray temperature was set at 4°C. Ultimately, the 10 aconitines were all determined within 9 min (Figure 2).

**TABLE 1:** Ion pairs and the detailed parameters in MRM mode.

Components	Retention time (min)	Q1	Q3	Time (ms)	DP (V)	CE (V)
Aconine	0.70	500.1	450.4	25	100	48
Mesaconine	0.70	486.1	436.0	25	100	49
Hypaconine	0.70	470.2	438.4	25	120	44
Songorine	1.13	358.1	340.1	25	129	36
Benzoylmesaconitine	1.17	590.0	540.1	25	120	50
Benzoylaconitine	1.42	604.1	554.2	25	124	50
Berberine	1.54	336.0	320.1	25	115	41
Benzoylhypaconitine	1.58	574.1	542.0	25	120	45
Mesaconitine	2.96	632.4	572.1	25	120	47
Hypaconitine	3.44	616.1	556.2	25	80	44
Indaconitine	3.55	630.2	570.1	25	80	47

### 3.2 Results of Method Validation

Representative chromatograms of blank rat plasma, corresponding spiked plasma, and plasma samples from rats 45 min after oral administration are shown in Figure 2, indicating good specificity and no interference from endogenous substances and metabolites in the experiment. In blank plasma, all 10 aconitines were not detected. The calibration curve of the target components exhibited good linearity in the range of 0.0156–125 ng/ml, with *R* ^2^ > 0.99 (Table 2). The LOD and LOQ of the target components were of 0.002–0.032 ng/ml and 0.006–0.095 ng/ml, respectively. For most components, the inter-day and intra-day precision with RSD were between 80 and 120% (Table 3) and were stable in short-term, long-term, and freeze-thaw experiments with RSD less than 10% (Table 4). The extraction recoveries and matrix effects for most components were in the range of 85–115% (Table 5), indicating that the pretreatment method met the requirements and the matrix had no significant effect on the target components. Generally, the quantification results were deemed accurate and reliable. Since its many measurement points of aconitine, a major diester alkaloid in raw Fuzi, were significantly below the lower limit of the linear calibration range of 0.125 ng/ml, it would be difficult to determine its concentration in real plasma samples. Therefore, the subsequent comparative pharmacokinetic study of aconitine was not carried out in this study.

**TABLE 2:** Linearity, LOD, and LOQ of target components.

Components	Calibration equation	*R* ^2^	Linearity range (ng/ml)	LOD (ng/ml)	LOQ (ng/ml)
Mesaconitine	*Y* = 0.012*X*−3.44e^−5^	0.9974	0.0156–31.3	0.0020	0.0059
Indaconitine	*Y* = 0.0271*X*+1.57e^−4^	0.9985	0.0625–31.3	0.0159	0.0477
Hypaconitine	*Y* = 0.0171*X*+1.53e^−3^	0.9995	0.125–62.5	0.0318	0.0953
Benzoylaconitine	*Y* = 0.00591*X*+0.14e^−3^	0.9930	0.0625–31.3	0.0079	0.0238
Benzoylmesaconitine	*Y* = 0.00469*X*+5.87e^−4^	0.9962	0.0313–62.5	0.0040	0.0119
Benzoylhypaconitine	*Y* = 0.0112*X*+4.74e^−4^	0.9960	0.0156–62.5	0.0020	0.0059
Aconine	*Y* = 0.00317*X*−3.79e^−5^	0.9913	0.0625–7.81	0.0159	0.0477
Mesaconine	*Y* = 0.00333*X*+8.12e^−4^	0.9964	0.0625–125	0.0079	0.0238
Hypaconine	*Y* = 0.00835*X*+0.01e^−1^	0.9956	0.0625–31.3	0.0079	0.0238
Songorine	*Y* = 0.0176*X*+6.58e^−4^	0.9956	0.0625–62.5	0.0079	0.0238

**TABLE 3:** Precision of target compounds.

Compound	Spiked (ng/ml)	Inter-day precision			Intra-day precision		
		Mean (ng/ml)	RSD (%)	Accuracy (%)	Mean (ng/ml)	RSD (%)	Accuracy (%)
Mesaconitine	0.5	0.47	7.72	94.33	0.46	2.08	91.17
	2	2.17	6.40	108.50	2.00	2.76	99.83
	20	21.17	4.23	105.83	20.00	1.50	100.00
Indaconitine	0.5	0.57	2.32	113.28	0.57	0.34	114.80
	2	1.71	5.14	85.49	1.60	2.86	80.16
	20	16.73	5.54	83.66	15.90	2.86	79.49
Hypaconitine	0.5	0.59	8.11	118.36	0.59	0.79	117.74
	2	1.80	3.92	90.04	1.63	1.86	81.70
	20	19.41	5.75	97.05	18.83	0.61	94.15
Benzoylaconitine	0.5	0.47	7.75	94.09	0.48	4.61	95.84
	2	2.28	1.12	113.96	2.13	1.84	106.56
	20	21.08	8.41	105.38	20.77	1.32	103.84
Benzoylmesaconitine	0.5	0.58	2.26	115.31	0.57	4.18	113.31
	2	1.72	5.51	85.81	1.68	2.56	83.90
	20	20.23	7.15	101.14	18.33	3.98	91.67
Benzoylhypaconitine	0.5	0.45	2.94	90.00	0.42	3.88	84.50
	2	2.16	4.70	108.16	2.03	1.59	101.33
	20	21.89	5.20	109.44	20.95	0.71	104.75
Aconine	0.5	0.57	4.98	113.53	0.59	5.64	117.85
	2	1.86	12.01	92.89	1.62	3.52	81.15
	5	4.86	7.46	97.14	4.77	2.18	95.31
Mesaconine	0.5	0.61	16.76	122.50	0.56	3.36	111.83
	2	2.19	3.20	109.26	2.12	12.80	106.02
	20	21.33	4.92	106.67	21.20	4.72	106.00
Hypaconine	0.5	0.56	8.90	112.97	0.54	1.47	107.06
	2	2.20	9.06	110.02	1.94	2.84	96.85
	20	21.79	4.69	108.96	21.36	0.71	106.82
Songorine	0.5	0.43	4.62	86.50	0.43	0.34	85.33
	2	2.18	3.05	109.17	2.17	3.33	108.67
	20	21.87	2.68	109.33	22.03	1.46	110.17

**TABLE 4:** Stability of target compounds.

Compound	Spiked ng/mL	Short-term		Long-term		3 times freeze-thaw	
		Mean ± SD (ng/ml)	RSD (%)	Mean ± SD (ng/ml)	RSD (%)	Mean ± SD (ng/ml)	RSD (%)
Mesaconitine	0.5	0.47 ± 0.04	7.63	0.46 ± 0.01	1.90	0.45 ± 0.01	2.11
	2	2.16 ± 0.15	6.98	2.01 ± 0.08	3.89	2.05 ± 0.08	3.70
	20	21.03 ± 1.02	4.86	20.13 ± 0.51	2.55	20.43 ± 0.38	1.85
Indaconitine	0.5	0.57 ± 0.01	0.92	0.57 ± 0.01	2.30	0.56 ± 0.01	1.97
	2	1.70 ± 0.10	5.92	1.61 ± 0.05	3.09	1.63 ± 0.07	4.07
	20	16.77 ± 0.90	5.34	15.86 ± 0.39	2.43	15.96 ± 0.41	2.58
Hypaconitine	0.5	0.59 ± 0.05	8.07	0.59 ± 0.01	1.41	0.58 ± 0.03	5.54
	2	1.78 ± 0.10	5.70	1.65 ± 0.06	3.64	1.72 ± 0.11	6.68
	20	19.44 ± 1.09	5.63	18.80 ± 0.13	0.70	18.75 ± 0.04	0.23
Benzoylaconitine	0.5	0.48 ± 0.02	4.95	0.47 ± 0.03	7.45	0.46 ± 0.02	4.41
	2	2.25 ± 0.08	3.42	2.16 ± 0.8	3.81	2.21 ± 0.11	4.94
	20	21.30 ± 1.67	7.85	20.55 ± 0.27	1.34	20.33 ± 0.53	2.60
Benzoylmesaconitine	0.5	0.58 ± 0.01	2.54	0.57 ± 0.02	4.15	0.58 ± 0.01	2.26
	2	1.73 ± 0.09	5.22	1.67 ± 0.04	2.25	1.65 ± 0.02	1.44
	20	19.92 ± 1.58	7.94	18.64 ± 1.25	6.72	18.99 ± 1.03	5.42
Benzoylhypaconitine	0.5	0.45 ± 0.01	3.22	0.42 ± 0.02	4.50	0.43 ± 0.02	4.73
	2	2.14 ± 0.13	6.02	2.05 ± 0.05	2.25	2.09 ± 0.03	1.54
	20	21.67 ± 1.29	5.97	21.16 ± 0.32	1.52	21.21 ± 0.27	1.26
Aconine	0.5	0.55 ± 0.04	6.75	0.55 ± 0.03	5.19	0.53 ± 0.02	3.54
	2	1.70 ± 0.11	6.65	1.73 ± 0.28	16.39	1.73 ± 0.24	13.63
	5	4.82 ± 0.36	7.49	4.72 ± 0.09	1.95	4.51 ± 0.18	4.02
Mesaconine	0.5	0.57 ± 0.07	13.05	0.59 ± 0.04	6.25	0.58 ± 0.10	18.00
	2	2.24 ± 0.11	4.83	2.15 ± 0.12	5.79	2.22 ± 0.04	1.88
	20	21.63 ± 1.16	5.36	20.90 ± 0.61	2.91	20.93 ± 0.55	2.63
Hypaconine	0.5	0.55 ± 0.05	8.89	0.55 ± 0.03	5.29	0.54 ± 0.04	7.17
	2	2.07 ± 0.12	5.66	2.07 ± 0.15	7.23	2.10 ± 0.26	12.32
	20	21.64 ± 1.05	4.85	21.52 ± 0.30	1.41	21.26 ± 0.53	2.50
Songorine	0.5	0.42 ± 0.01	2.45	0.44 ± 0.02	3.50	0.43 ± 0.02	4.69
	2	2.19 ± 0.07	3.20	2.17 ± 0.07	3.07	2.18 ± 0.08	3.57
	20	21.97 ± 0.67	3.03	21.93 ± 0.15	0.70	21.73 ± 0.47	2.17

**TABLE 5:** Recovery and matrix effect of PK method.

Compound	Spiked ng/mL	Recovery		Matrix effect	
		Mean (%)	RSD (%)	Mean (%)	RSD (%)
Mesaconitine	0.5	105.22	2.96	90.38	3.81
	2	104.61	4.49	87.66	4.92
	20	109.87	2.19	90.09	7.72
Indaconitine	0.5	101.55	4.56	97.81	8.15
	2	105.44	6.74	95.51	5.93
	20	110.96	3.15	97.29	7.00
Hypaconitine	0.5	109.50	3.08	106.81	3.93
	2	107.67	4.24	108.32	7.45
	20	109.67	2.17	86.04	7.05
Benzoylaconitine	0.5	111.88	9.46	95.65	3.69
	2	109.76	7.71	110.54	9.95
	20	106.18	2.43	97.94	8.38
Benzoylmesaconitine	0.5	103.44	4.52	83.35	2.12
	2	102.78	4.95	88.45	3.03
	20	108.75	4.82	109.16	5.42
Benzoylhypaconitine	0.5	107.03	2.10	90.72	0.84
	2	104.04	2.96	105.93	7.47
	20	105.69	2.93	94.86	5.77
Aconine	0.5	104.41	7.08	87.82	6.21
	2	106.76	4.91	105.70	13.84
	5	100.69	5.53	116.04	10.92
Mesaconine	0.5	101.67	10.84	110.47	14.64
	2	105.51	14.35	108.25	7.43
	20	109.49	7.67	108.84	9.95
Hypaconine	0.5	98.20	9.88	97.60	11.17
	2	100.90	8.22	83.20	7.74
	20	100.82	11.04	116.22	9.86
Songorine	0.5	83.24	12.37	87.25	4.35
	2	85.17	2.16	102.45	7.06
	20	95.18	1.75	93.01	7.81

### 3.3 Comparative Pharmacokinetic Study

**FIGURE 3:**
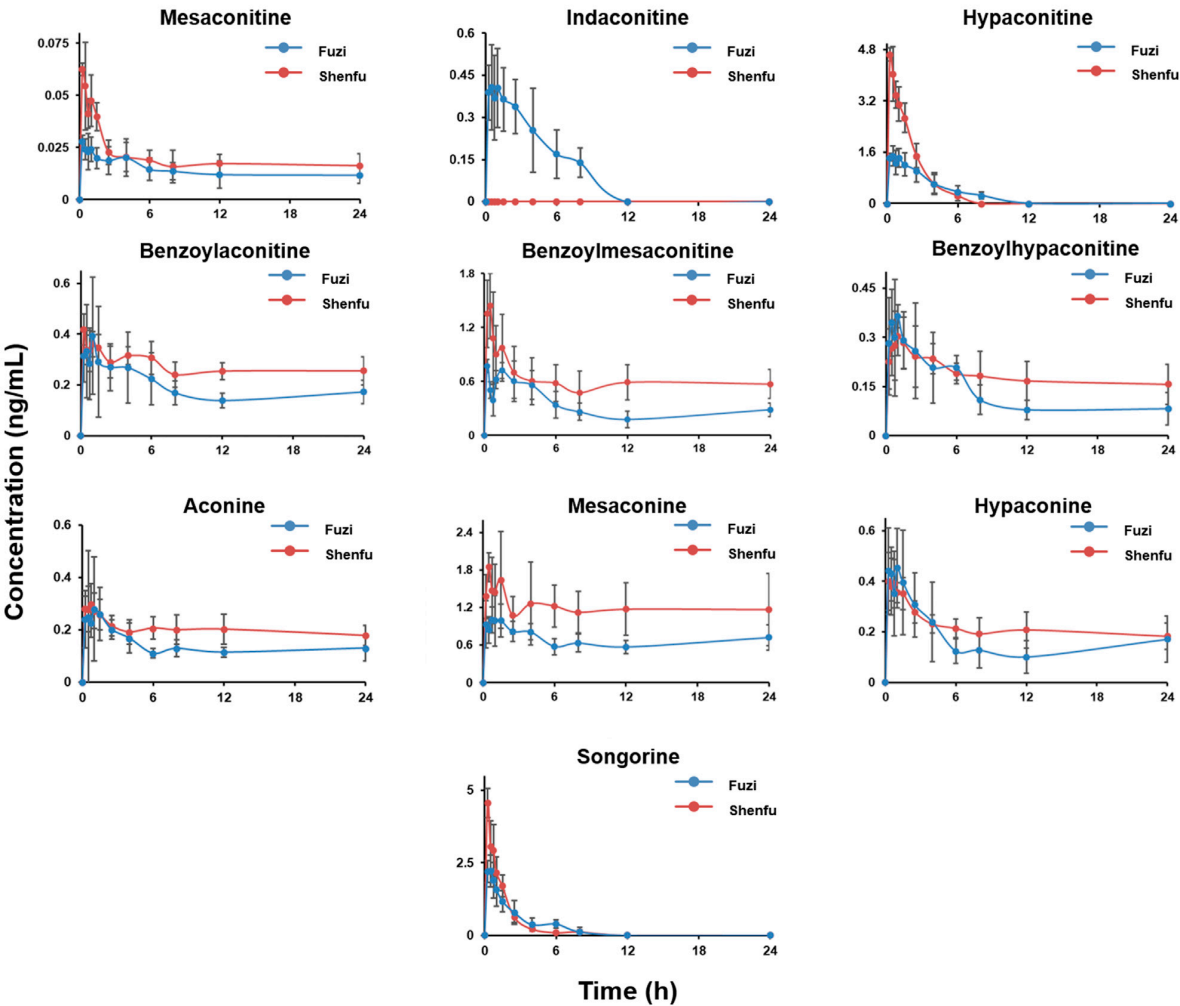
The concentration-time profile of aconitines after oral administration of Fuzi and Shenfu. The dosage of Fuzi is all 2 g/kg in two groups.

As can be seen from Figure 3, in terms of the overall trend, a distinct peak shape is visible in both the Fuzi and Shenfu groups. In the plasma concentration-time curve of the diester alkaloids and monoester alkaloids, double peaks were evident (e.g., benzoylaconitine). This has been similar in other studies (Liu et al., 2014; Li et al., 2016; Zhang et al., 2020). This biabsorption phenomenon may come from multiple-sites absorption and enterohepatic circulation (Zhang et al., 2020). In Fuzi and Shenfu decoctions, the monoester alkaloids benzoylaconitine, benzoylmesaconitine, and benzoylhypaconitine were the main class of components. However, the monoester alkaloids, non-ester alkaloids, and hypaconitine were the main components *in vivo*. This compatibility had a significant decrease of *in vivo* exposure of an active diester alkaloid indaconitine (Yu et al., 2021). This may be because some components of ginseng prevent the dissolution of indaconitine in Shenfu decoction. The short *T*_max_ and *T*_1/2_ of the aconitines (Table 6) exhibited the distinct characteristics of fast absorption and rapid elimination after oral administration of the extract of Fuzi (Song et al., 2015; Xu et al., 2017) and Shenfu. In contrast, the long *T*_1/2_ of some components (e.g., benzoylmesaconitine in the Shenfu group) may be since half of the *C*_max_ had not yet been reached at the end of the 24 h experiment. It is noteworthy that in this study, the minor diester alkaloid yunaconitine was not detected in Fuzi and Shenfu decoctions and the rat plasma.

**TABLE 6:** Comparison with the pharmacokinetic parameters of aconitines in Fuzi before and after compatibility of ginseng (mean ± SD, *n* = 6).

Components	Fuzi					Shenfu					Relative bioavailability/(*F*_*rel*_, %)
	*T*_1/2_/h	*AUC*_0-t_/(ng/mL·min)	*MRT*_0-t_/(ng/mL·min)	*T*_max_/h	*C*_max_/(ng/ml)	*T*_1/2_/h	*AUC*_0-t_/(ng/mL·min)	*MRT*_0-t_/(ng/mL·min)	*T*_max_/h	*C*_max_/(ng/ml)	
Mesaconitine	21.74 ± 3.85	0.33 ± 0.07	10.73 ± 1.29	0.50 ± 0.09	0.02 ± 0.00	89.71 ± 9.32***	0.46 ± 0.07**	10.58 ± 1.80	0.50 ± 0.07	0.05 ± 0.00	144.00 ± 35.83
Indaconitine	2.36 ± 0.35	2.29 ± 0.62	3.88 ± 0.58	0.50 ± 0.10	0.41 ± 0.15	-	-	-	-	-	-
Hypaconitine	2.07 ± 0.53	6.16 ± 0.64	3.29 ± 0.50	0.50 ± 0.10	1.48 ± 0.34	2.65 ± 0.50	8.93 ± 1.10***	1.94 ± 0.26**	0.50 ± 0.06	4.04 ± 0.16***	146.12 ± 22.30
Benzoylaconitine	9.28 ± 0.18	4.47 ± 0.54	10.79 ± 2.16	1.00 ± 0.07	0.39 ± 0.22	80.39 ± 16.01	6.43 ± 0.90**	11.59 ± 1.39	1.00 ± 0.12	0.37 ± 0.05	144.94 ± 23.37
Benzoylmesaconitine	6.48 ± 1.03	7.56 ± 0.91	10.02 ± 0.80	1.50 ± 0.21	0.83 ± 0.07	524.59 ± 99.67***	14.55 ± 3.64**	11.38 ± 1.71	0.50 ± 0.06***	1.44 ± 0.36**	195.96 ± 53.55
Benzoylhypaconitine	4.65 ± 0.65	3.11 ± 0.53	8.79 ± 1.41	1.00 ± 0.11	0.36 ± 0.03	32.55 ± 5.35***	4.42 ± 0.51**	10.91 ± 1.31*	1.00 ± 0.15	0.30 ± 0.05*	147.07 ± 37.80
Aconine	-	3.30 ± 0.43	11.05 ± 2.80	1.00 ± 0.04	0.28 ± 0.12	89.78 ± 14.78	4.78 ± 1.04*	11.49 ± 1.42	0.75 ± 0.08***	0.30 ± 0.07	147.42 ± 43.85
Mesaconine	124.13 ± 18.62	16.31 ± 2.61	11.74 ± 2.00	0.75 ± 0.09	1.00 ± 0.16	304.61 ± 57.87***	28.64 ± 3.44***	11.81 ± 1.65	0.50 ± 0.10**	1.85 ± 0.22***	177.17 ± 17.37
Hypaconine	18.31 ± 2.01	3.98 ± 1.35	10.61 ± 3.60	1.00 ± 0.12	0.45 ± 0.17	75.83 ± 13.64	5.15 ± 0.77	10.98 ± 2.74	0.75 ± 0.07**	0.39 ± 0.11	141.60 ± 47.45
Songorine	2.17 ± 0.31	5.56 ± 0.67	2.77 ± 0.66	0.50 ± 0.07	2.22 ± 0.53	2.56 ± 0.53	5.69 ± 0.71	2.00 ± 0.38*	0.50 ± 0.02	3.07 ± 0.33**	103.05 ± 20.96

*p < 0.05; **p < 0.01; ***p < 0.001.

The minimum toxic doses of mesaconitine and hypaconitine in humans have been reported as 0.0035 and 0.0162 mg/kg, respectively (Qiu et al., 2020b), which can be converted to 21.88 and 101.25 μg/kg in rats (Huang et al., 2004). The doses of the two diester alkaloids in this experiment were 0.34 μg/kg for mesaconitine and 11.86 μg/kg for hypaconitine in Fuzi decoction, and 1.80 μg/kg for mesaconitine and 87.03 μg/kg for hypaconitine in Shenfu decoction. After the application of our developed toxicity prediction method (Qiu et al., 2020a; Qiu et al., 2020b), it was found that the *in vivo* holistic weighted toxicity (HWT) value was less than 1, indicating all alkaloids were below the minimum toxic doses. Therefore, the three diester alkaloids showed no toxicity but only medicinal effects under the present conditions. As Table 6 shown, *in vivo* exposure of Fuzi and Shenfu groups, the hypaconitine and benzoylmesaconitine are representatively active components of the diester alkaloid and monoester alkaloid, respectively. As for compatibility, the mechanism of drug interactions is complicated. This study attempts to clarify these interactions between aconitines and ginsenosides. Comparing the drug concentration-time curves of Fuzi group and Shenfu group (Figure 3), it was found that in some alkaloids with higher absorption (e.g., hypaconitine and songorine), the Shenfu group decreased to plateau more quickly than the Fuzi group. However, their *AUC*_0-t_ values of Shenfu groups (5.69 for songorine, 8.93 for hypaconitine) were higher than those of Fuzi group (5.56 for songorine, 6.16 for hypaconitine), indicating the hypaconitine and songorine in the Shenfu group was significantly faster than those of Fuzi group in the elimination phase. The main reason might be that the ginsenoside Rg_1_ could promote absorption of aconitines (Xu et al., 2020) and up-regulate *in vivo* expression of CYP450 for accelerating the metabolism of hypaconitine and songorine (Li et al., 2019). The exposure concentrations of other aconitines were higher in Shenfu group, especially for the diester alkaloid (i.e., mesaconitine), monoester alkaloids (i.e., benzoylaconitine, benzoylmesaconitine, and benzoylhypaconitine) (He et al., 2015; Xie et al., 2021), and non-ester alkaloids (i.e., aconine and mesaconine). Their *T*_1/2_ and *AUC*_0-t_ would be significantly increased in Shenfu group (Table 6), which may be caused by the inhibitory of the P-glycoprotein (P-gp)-mediated aconitines efflux by *in vivo* metabolites of ginsenosides (Chen et al., 2009; Tang et al., 2012; Li et al., 2014). These phenomena were consistent with Xu’s study (Xu et al., 2020). Among all exposed components, songorine, a non-ester alkaloid with good anti-arrhythmic effects (Dzhakhangirov et al., 1997; Khan et al., 2018) and cardioprotection efficacy (Li et al., 2021), showed a larger *C*_max_ within 1 h, which could effectively eliminate the potential cardiotoxicity of the diester alkaloids (e.g., mesaconitine and hypaconitine). In short, the compatibility of Fuzi and ginseng could significantly increase the *in vivo* exposure of the active ingredients.

## 4 Conclusion

An HPLC-MS-based method was developed for the quantification of 10 aconitines in rat plasma within 9 min, with the LOD of 0.002–0.032 ng/ml and LOQ of 0.006–0.095 ng/ml. A comparative pharmacokinetic study was conducted in SD rats orally administered with the Fuzi and Shenfu decoction. Under safe dosage, it was found that for most alkaloids, including diester type alkaloids (mesaconitine and hypaconitine) and monoester alkaloids (benzoylaconitine, benzoylmesaconitine, and benzoylhypaconitine), were exposed more in Shenfu group (*AUC*_0-t_ were 0.46–14.55 ng/mlmin) than in Fuzi group (*AUC*_0-t_ were 0.33–7.56 ng/mlmin). Except for the hypaconitine, other components were metabolized more slowly in Shenfu group than in Fuzi group. Therefore, the compatibility of Fuzi and ginseng could significantly increase the bioavailability (103.05–195.96%) and efficiency of active components *in vivo*. songorine containing a potential anti-cardiotoxicity ability showed a larger *C*_max_. Ultimately, the hypaconitine, benzoylmesaconitine, and songorine could be considered as the main active components in Shenfu oral prescriptions. This study aims to achieve clinical “efficacy enhancement” and lay the foundation for the scientific design of new Shenfu oral prescriptions.

## Data Availability

The original contributions presented in the study are included in the article/Supplementary Material, further inquiries can be directed to the corresponding authors.
